# Transcatheter edge-to-edge repair for arcade-like mitral apparatus: expanding treatment options and assessing clinical outcome

**DOI:** 10.3389/fcvm.2025.1571818

**Published:** 2025-09-01

**Authors:** Qinglan Shu, Yi Wang, Cong Lu, Lixue Yin, Yan Deng, Yun Xu, Yi Zhou, Tiantian Luo, Sijia Wang, Jie Zeng

**Affiliations:** ^1^ Department of Cardiovascular Ultrasound & Noninvasive Cardiology, Sichuan Provincial People's Hospital, University of Electronic Science and Technology of China, Chengdu, China; ^2^ Ultrasound Medicine and Computational Cardiology Key Laboratory of Sichuan Province, Sichuan Provincial People's Hospital, University of Electronic Science and Technology of China, Chengdu, China; ^3^ Department of Cardiology, Structural Heart Disease Center, Sichuan Provincial People's Hospital, University of Electronic Science and Technology of China, Chengdu, China

**Keywords:** echocardiography, mitral regurgitation, heart failure, mitraI arcade, transcatheter mitraI repair

## Abstract

**Background:**

The arcade-like mitral apparatus is a rare congenital anomaly characterized by complex subvalvular pathology, often resulting in mitral dysfunction. These anatomical complexities make conventional surgical interventions particularly challenging, especially for elderly high-risk patients. For these patients, less invasive options like Transcatheter Edge-to-Edge Repair (TEER) present a promising alternative, addressing both anatomical challenges and procedural risks.

**Aims:**

To assess the feasibility, procedural success, safety, and clinical outcomes of TEER in patients with isolated severe MR due to arcade-like mitral apparatus.

**Methods:**

This case series involved four high-risk patients with isolated severe mitral regurgitation (MR) secondary to arcade-like mitral apparatus treated with TEER between August 2022 and August 2023. Each patient was evaluated by a multidisciplinary Heart Team to ensure optimal selection and procedural planning. Detailed anatomical assessment using advanced imaging techniques, was performed to customize the approach and ensure procedural success. The MitraClip XTR device was employed in all cases, with careful attention to patient-specific anatomical challenges.

**Results:**

TEER was successfully performed in all patients, with immediate and sustained reductions in MR severity. At the one-year follow-up, all patients demonstrated improved cardiac function, an increase in New York Heart Association (NYHA) functional class, and a reduction in Borg dyspnea scores. Significant improvements in myocardial mechanics and work parameters were observed. Global Longitudinal Strain (GLS) improved significantly compared to baseline. The Global Work Index (GWI), Global Constructive Work (GCW), Global Pressure-Volume Work (GPW), Global Systolic Constructive Work (GSCW) and Global Work Efficiency (GWE) also showed a marked increase.

**Conclusion:**

TEER represents a promising, minimally invasive option for managing severe MR due to arcade-like mitral apparatus in high-risk patients. This case series underscores TEER's potential to offer significant symptom relief and improved hemodynamics, presenting a new therapeutic perspective for treating isolated MR in this anatomically challenging condition. Further large-scale studies are warranted to validate these findings and establish TEER's role in broader clinical practice.

## Introduction

The arcade-like mitral apparatus, also referred to as anomalous mitral arcade, is a rare congenital anomaly characterized by a complex subvalvular architecture, including thickened and shortened chordae tendineae as well as hypertrophied papillary muscles abnormally displaced superiorly (1). This anatomical configuration often results in a dual pathology of mitral stenosis and regurgitation, complicating conventional therapeutic approaches (2). Historically, the most commonly reported treatment approach for this condition has been surgical mitral valve replacement (3).

For patients with isolated severe MR without significant stenosis, the delayed onset of symptoms introduces unique challenges. As symptoms typically emerge later in life, the risks associated with open-heart surgery increase significantly with advanced age. Compensatory mechanisms often mask symptoms, and by the time intervention is required, patients may have accumulated substantial surgical risks due to frailty and comorbidities.

The arcade-like mitral apparatus presents substantial challenges for TEER due to its intricate subvalvular anatomy and abnormal leaflet morphology. Achieving sufficient leaflet coaptation is particularly difficult in this subset of patients. Despite these obstacles, advancements in catheter-based technologies and imaging techniques have made TEER a potential option for such patients (4). The current study aims to assess the feasibility, safety, and clinical outcomes of TEER in patients with isolated severe MR secondary to arcade-like mitral apparatus, thereby contributing to our understanding of its therapeutic potential in this high-risk cohort.

## Methods

### Patient selection

**Figure 1:**
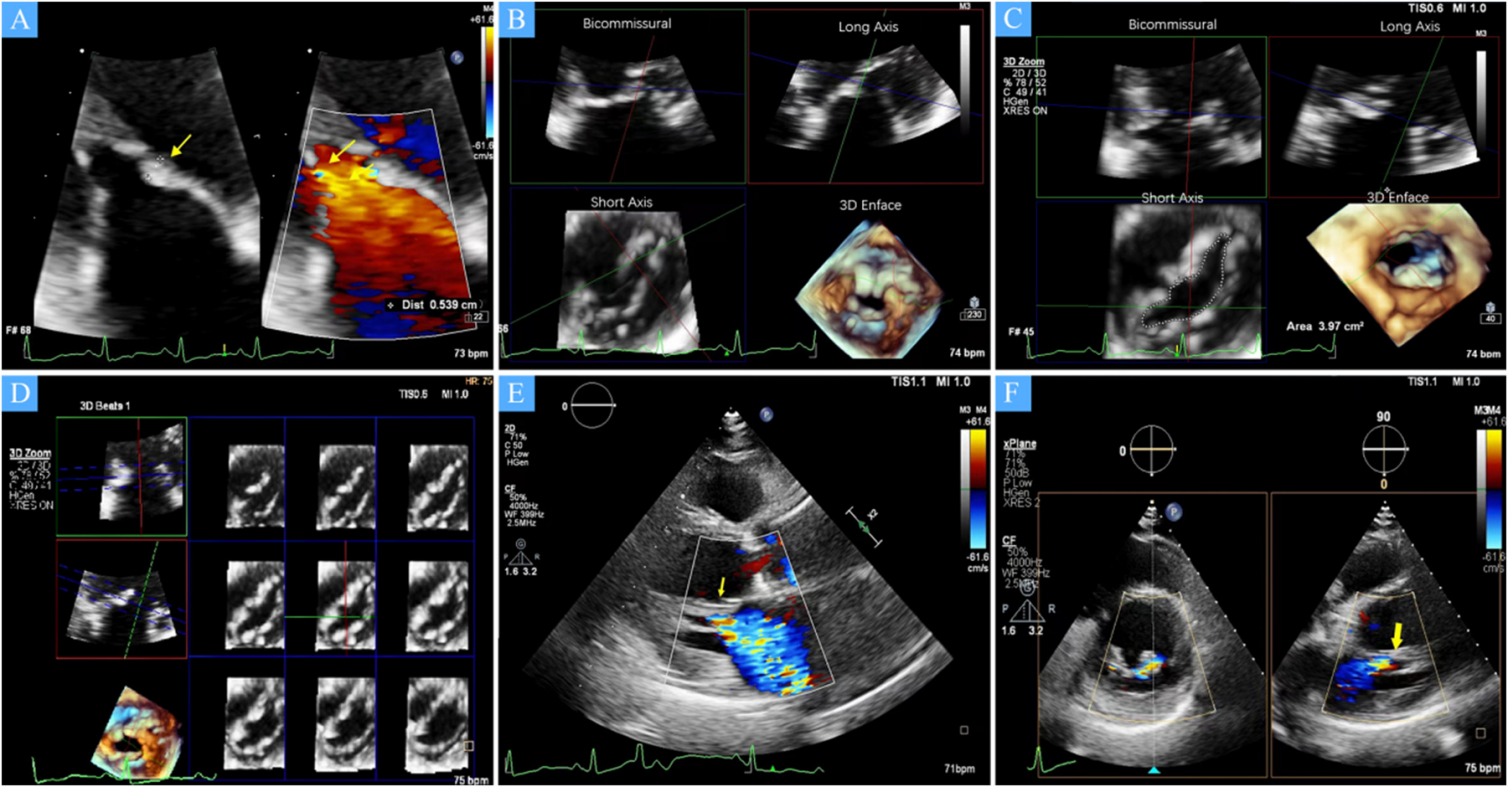
**(A)** A zoomed image of the mitral valve shows the thickness of the anterior leaflet in this area is approximately 5 mm (yellow arrow), with short, thick, and poorly differentiated chordae that directly connect the papillary muscles to the anterior leaflet, and narrow spaces between the abnormal chordae (yellow star). **(B)** Multiview 3D transthoracic echocardiography (3D TTE) illustrates short, thick, and poorly differentiated chordae with direct connection to the anterior leaflet. **(C)** Multiview 3D TTE reveals a mitral valve area of 3.97 cm^2^ without commissural fusion, effectively ruling out rheumatic valve disease. **(D)** Multislice 3D TTE shows uneven thickening of the mitral valve without commissural fusion and calcification. **(E)** The left ventricular long-axis view on transthoracic echocardiography (TTE) demonstrates a direct connection between the papillary muscle and the anterior mitral leaflet, with significant mitral regurgitation during systole (arrow). **(F)** TTE biplane short-axis view of the left ventricle shows a direct connection between the papillary muscle and the anterior mitral leaflet (yellow arrow).

This case series included four patients who underwent TEER for isolated severe MR secondary to arcade-like mitral apparatus between August 2022 and August 2023. All patients were evaluated by a multidisciplinary Heart Team composed of interventional cardiologists, cardiac surgeons, and imaging specialists. These four patients were selected from over 2,000 cases of isolated severe mitral regurgitation diagnosed during the study period, underscoring the careful selection process of identifying anatomically challenging and high-risk cases. Inclusion criteria involved patients with severe MR, distinctive anatomical features consistent with arcade-like mitral apparatus (Figure 1), and high surgical risk as assessed by the Society of Thoracic Surgeons Predicted Risk of Mortality (STS-PROM) score. Despite repeated hospital admissions for the relapsing condition, all four patients declined open-heart surgery. The Heart Team assessed each patient as high-risk, and after thorough discussions with the patients and their families, the decision was made to proceed with TEER as the preferred treatment option. This multidisciplinary approach ensured that the treatment plan considered both the anatomical complexities and the patients' preferences.

The first case involved a 70-year-old female patient with a 4-year history of fatigue, paroxysmal nocturnal dyspnea. Over the preceding five months, her exercise tolerance had significantly decreased, and her symptoms had become progressively refractory to guideline-directed medical therapy. On admission, the patient's blood pressure was 94/65 mmHg, with noted bilateral lower extremity edema. Electrocardiography demonstrated atrial fibrillation and complete left bundle branch block. Laboratory analysis revealed a markedly elevated N-terminal pro-B-type natriuretic peptide (NT-proBNP) level of 11,268 pg/ml. Coronary angiography ruled out significant coronary artery disease, excluding ischemic heart disease as the underlying etiology. Computed tomography (CT) revealed bilateral pleural effusions. The STS-PROM score was approximately 7%, This elevated risk is driven by her advanced age, severe heart failure presentation, moderately reduced ejection fraction (EF) (35%), and other comorbid factors, such as atrial fibrillation. In light of her persistent symptoms and absence of coronary artery disease, the multidisciplinary Heart Team recommended TEER. The decision was guided by the anatomical and clinical challenges posed by her mitral valve pathology and the elevated surgical risk associated with conventional open-heart surgery in this patient.

The second case involved a 66-year-old male who presented with dyspnea and a prominent 4/6 systolic murmur auscultated at the cardiac apex. He had a history of chronic heart failure, which had been managed with optimized medical therapy. On examination, his blood pressure was 116/70 mmHg, and bilateral lower extremity edema was present. Electrocardiography demonstrated sinus rhythm with broadening of the P wave and nonspecific ST-segment changes. Laboratory findings showed an elevated NT-proBNP level of 1, 194 pg/ml. Coronary angiography identified only clinically insignificant coronary artery stenosis, excluding any significant ischemic contributions to his mitral regurgitation. CT revealed bilateral pleural effusions. Given the patient's mild cachexia (body mass index: 17.5 kg/m^2^) and an elevated STS-PROM score exceeding 6% for mitral valve repair, the Heart Team elected to proceed with TEER. This approach was chosen to mitigate surgical risks while effectively addressing the patient's severe mitral regurgitation.

The third case was a 54-year-old female presenting with persistent exertional dyspnea, which was unresponsive to optimized guideline-directed medical therapy. The patient has a long-standing history of hypertension managed with chronic antihypertensive therapy, with documented blood pressure readings reaching 160/94 mmHg. Her electrocardiography demonstrated sinus rhythm with a prominent P wave terminal force in the V1 lead. Laboratory investigations revealed an elevated NT-proBNP level of 1,008 pg/ml. Coronary computed tomography angiography excluded significant coronary artery disease, confirming the absence of obstructive coronary pathology. The STS-PROM was approximately 6%, reflecting a high-risk clinical profile marked by advanced heart failure (NYHA class III with recent acute decompensation), a moderately reduced left ventricular EF of 32%, and multiple comorbidities, including hypertension, diabetes mellitus (Fasting Plasma Glucose: 11.82 mmol/L), chronic bronchitis, and renal dysfunction: (Creatinine1.46 mg/dl). The decision to proceed with TEER was based on her ongoing symptoms despite optimal medical management, unique mitral valve anatomical features, and the potential benefits of a minimally invasive intervention. This was selected to address her significant mitral regurgitation while minimizing the risks associated with conventional surgery.

The fourth case involved a 76-year-old male with a decade-long history of recurrent dyspnea, recently exacerbated by a worsening cough over the past 20 days. His medical history included longstanding hypertension and a pacemaker implantation 10 years prior for third-degree atrioventricular block. On admission, transthoracic echocardiography demonstrated severe mitral regurgitation, marked left ventricular enlargement, and a mildly reduced left ventricular EF of 47%. The STS-PROM was approximately 6%.This elevated risk was attributable to the patient's severe heart failure (NYHA class III with acute decompensation), diabetes mellitus (Fasting Plasma Glucose: 14.88 mmol/L), status post pacemaker implantation, and renal dysfunction (Creatinine: 1.35 mg/dl). Coronary angiography revealed only non-obstructive coronary artery disease. Considering his complex clinical profile, including significant long-term cardiac comorbidities and severe mitral regurgitation, TEER was determined to be the most appropriate therapeutic strategy. This decision aimed to effectively alleviate his mitral regurgitation while minimizing the risks associated with more invasive surgical procedures, given the patient's advanced age and multiple comorbidities.

### Baseline characteristics

The study cohort consisted of four patients, two males and two females, with a mean age of 68 (54–76) years. All patients were in NYHA functional class III–IV at baseline. Echocardiographic assessment showed complex mitral valve anatomy, with thickened and shortened chordae tendineae and superiorly displaced hypertrophied papillary muscles (Figure 1). The left ventricular end-diastolic diameter (LVEDD) was 60.5 (57–72) mm, and the left ventricular EF was 36.5 (32–47)%, with further echocardiographic and volumetric details provided in Tables 1–3.

**Table 1:** Baseline clinical characteristics of the four study patients.

Baseline clinical characteries	Patient			
	1	2	3	4
Gender	female	male	female	male
Age	70	66	54	76
BSA	1.67	1.6	1.47	1.91
NYHA	IV	IV	Ⅲ	Ⅲ
NT-proBNP (pg/ml)	11,268	4,666	1,008	175
Blood pressure (mmHg)	94/65	116/70	93/70–160/94^a^	115/58
Heart rhythm	Paroxysmal atrial fibrillation	Sinus rhythm	Sinus rhythm	Sinus rhythm
HR (bpm)	68	72	70	69
Coronary	no significant stenosis	no significant stenosis	no significant stenosis	no significant stenosis
Heart failure medical treatment	GDMT >3 month	GDMT >3 month	GDMT >3 month	GDMT >3 month
STS-PROM score (%)	7	6	6	6

BSA, body-surface area; GDMT, guideline-directed medical therapy; NT-proBNP, N-terminal pro-B-type natriuretic peptide; NYHA, New York Heart Association; STS-PROM, Society of Thoracic Surgeons predicted risk of mortality; mmHg, millimetres of mercury.

^a^ The patient has a long-standing history of hypertension managed with chronic antihypertensive therapy, with documented blood pressure readings reaching 160/194 mm Hg.

**Table 2:** Echocardiographic findings.

Echocardiographic findings	Patient			
	1	2	3	4
EF (%)	35	38	32	47
LVEDV (ml)	256	193	104	84
LVEDVI (ml/m²)	174.15	115.57	65	44.21
MV orifice AL-PM Diam (mm)	42	39	31	31
MV orifice AP Diam (mm)	19	15	14	12
MV orifice aspect ratio	2.2	2.6	2.2	2.6
MV orifice area (cm^2^)	4.7	4.0	3.8	3.5
MV leaflets	thickening	uneven thickening	thickening	thickening
Short thickened chordae (diastolic chordae length <5 mm)	+	+	+	+
Enlarged and elongated papillary muscles	+	+	+	+
MV origin of regurgitation	central zone	lateral zone	central zone	central zone with a slight bias toward the medial

MV, mitral valve.

**Table 3:** The conventional echocardiographic parameters.

The conventional echocardiographic parameters	Pre MC	Group (median (range))	
		Post MC 3-day	Post MC 1-year
LA (mm)	51 (44–55)	47.5 (37–54)	44.5 (37–54)
LVEDD (mm)	61.5 (57–72)	57 (55–70)	53 (48–65)
LVEDV (ml)	148.5 (84–256)	127 (86–189)	93 (56–181)
LVESV (ml)	83.5 (45–164)	79 (34–144)	42 (25–98)
LVSV (ml)	65 (39–92)	48 (45–52)	51.5 (30–83)
LVEF (%)	36.5 (32–47)	38 (24–62)	54.5 (46–67)
HR (bpm)	69.5 (68–72)	64 (56–81)	67 (63–81)
SBP (mmHg)	115.5 (94–160)	116 (95–126)	112.5 (101–133)
DBP (mmHg)	68.5 (58–94)	69 (57–87)	68.5 (60–73)
MPG (mmHg)	3 (2–3)	3 (2–5)	3 (1–5)

HR, heart rate; SBP, systolic blood pressure; DBP, diastolic blood pressure; MPG, mean pressure gradient.

Detailed anatomical assessment was performed using multimodality echocardiography. In each case, patients underwent comprehensive two-dimensional transthoracic echocardiography (TTE) and transesophageal echocardiography (TEE) with three-dimensional capabilities for precise evaluation of the mitral valve anatomy and function. The echocardiographic studies were conducted on a high-end ultrasound system (GE Vivid E95, GE Healthcare). Myocardial strain and work indices were analyzed off-line using dedicated speckle-tracking software (EchoPAC, GE Healthcare) to derive global longitudinal strain (GLS) and pressure–strain loop-based myocardial work parameters. To contextualize these measurements, normal reference values for key parameters were noted: left ventricular EF: 60%, GLS: −20%, Global Work Index (GWI): 2,000 mmHg%, Global Constructive Work (GCW): 2,300 mmHg%, Global Wasted Work (GWW) <150 mmHg%, and Global Work Efficiency (GWE) >90% (5).

### Mitral regurgitation characteristics

All patients in this series presented with severe mitral regurgitation accompanied by reduced ventricular function, which was not the conventional ventricular-type functional mitral regurgitation (FMR). The restricted leaflet motion was due to the abnormal development of the mitral apparatus rather than tethering caused by ventricular dilation and chorda displacement. Notably, two patients exhibited central MR, in one patient, the regurgitant jet originated from the lateral region, whereas in another, it arose from the central with a slight bias toward the medial, suggesting heterogeneity in regurgitation origin and leaflet involvement. The mitral valve orifice was elliptical in shape, with an aspect ratio (defined as the ratio of the mitral orifice's long-axis length to its short-axis width on the en face view) greater than 2 (Table 2), which presented challenges for clip selection and positioning. Importantly, no evidence of commissural fusion, or involvement of other connective tissue disorders was found, helping to differentiate from rheumatic heart disease. The subvalvular chordae tendineae were notably thickened and shortened, but without evidence of adhesions or calcification, further ruling out rheumatic involvement. The impact of the clip on this type mitral valve orifice area also posed significant challenges.

### Procedural details

All procedures were performed under general anesthesia. To ensure a secure and stable leaflet grasp in these challenging scenarios, our heart team decided that the XTR device would be the most balanced choice, despite the usual preference for NT in restricted anatomies. This decision was made after thorough discussion and consideration of the unique anatomical aspects, leaflet length, valve orifice area, and baseline mitral transvalvular pressure gradient of these patients. We acknowledge the concern that the XTR's longer clip arms can exert greater tension on the leaflets, potentially leading to higher transmitral pressure gradients. Patient safety and hemodynamic performance were our top priorities. To mitigate the risk of mitral stenosis, we adopted a very cautious deployment technique. Specifically, the clip arms were closed gradually and not “over-closed,” as per best practices for the XTR device. By avoiding an overly tight grasp, we minimized excess tissue stress and preserved as much valve area as possible. As a result of this careful technique, the post-procedural mean mitral valve gradients remained ≤ 5 mmHg in all patients, even for the two cases with baseline mitral valve area (MVA) < 4.0 cm^2^, indicate that no significant mitral stenosis was induced by the clip, and thus the hemodynamic results were satisfactory.

Transseptal puncture was performed under fluoroscopic and three-dimensional transesophageal echocardiography (3D-TEE) guidance. Given the abnormal subvalvular anatomy, particular attention was paid to the narrow subvalvular space and leaflet morphology to achieve optimal leaflet capture. Multiplanar reconstruction (MPR) was employed to assist in the precise adjustment of the clip, thereby maximizing the coaptation surface area and minimizing residual MR. Procedural success was confirmed intraoperatively using TEE. Pre- and post-procedural left atrial pressure (LAP) and pulmonary artery systolic pressure (PASP) were measured using transcatheter techniques to comprehensively assess the hemodynamic impact of the intervention, providing a detailed evaluation of procedural effectiveness.

### Statistical analysis

Statistical analyses were performed using SPSS version 22.0. Data are presented as median (range). One-way analysis of variance (ANOVA) was performed to assess variations in the conventional echocardiographic parameters myocardial work and strain parameters at baseline, three days post-procedure, and one-year follow-up (only for straightforward presentation of data).

### Follow-up

TTE follow-up was conducted at 3 days post-procedure and 1 year post-procedure to evaluate the durability of the repair, including left ventricular function and MR severity.

## Results

### Procedural outcomes

**Figure 2:**
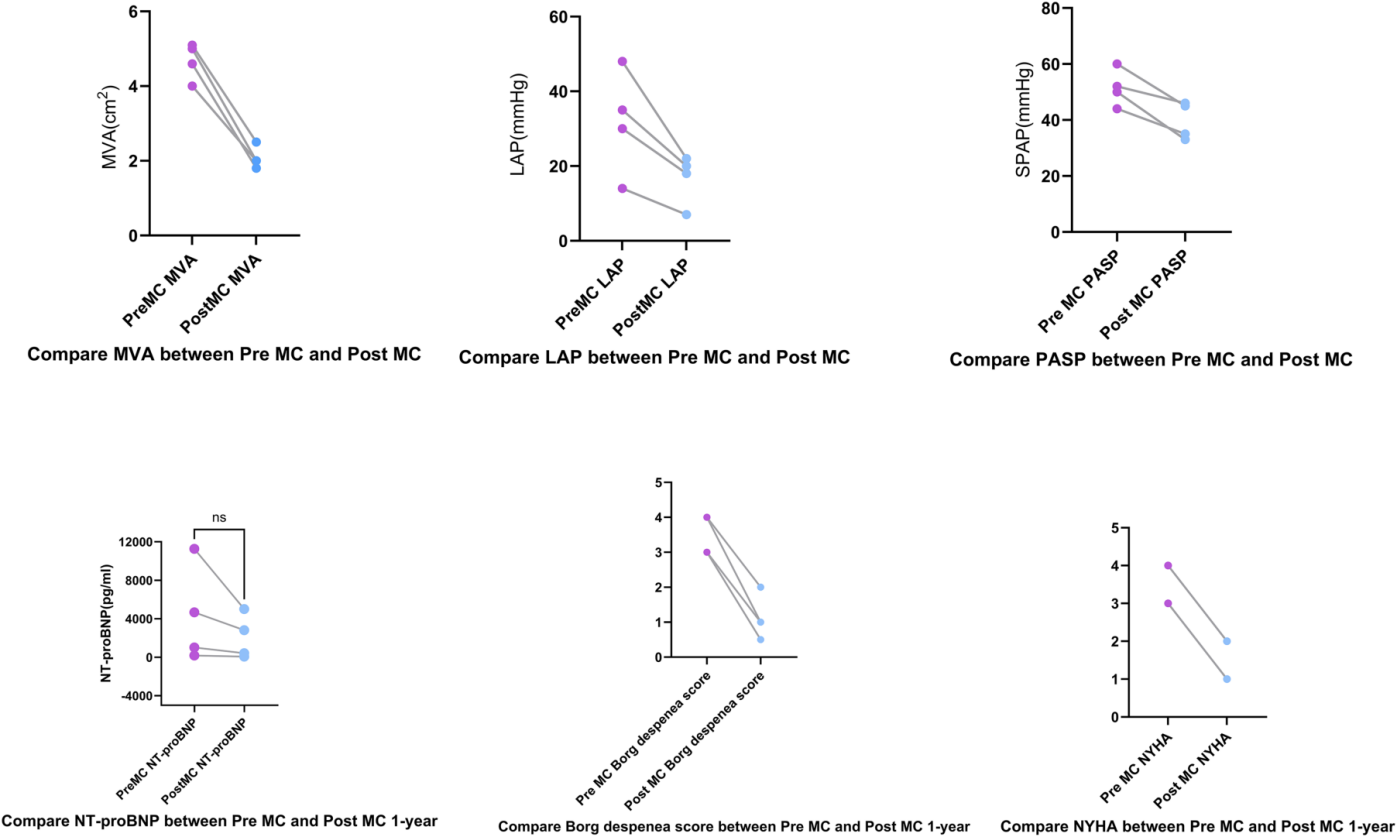
Compared to pre-MC, the left atrial pressure (LAP), mitral valve area (MVA), and pulmonary artery systolic pressure (PASP) decreased post-MC. At the 1-year follow-up, Borg dyspnea scores decreased, New York Heart Association (NYHA) functional class improved, and NT-proBNP showed a trend toward reduction.

TEER was successfully performed in all four cases, with immediate and significant reduction in MR severity. The mean transmitral gradient remained below 5 mmHg in all patients (Supplementary S1), with post-procedural MR reduced to mild. One patient required repositioning of the clip to optimize leaflet coaptation and reduce the transmitral gradient from an initial value of 6 mmHg. At the one-year follow-up, all patients demonstrated improved cardiac function, an increase in NYHA functional class, and a reduction in Brog dyspnea scores (Figure 2).

### Hemodynamic and clinical outcomes

**Figure 3:**
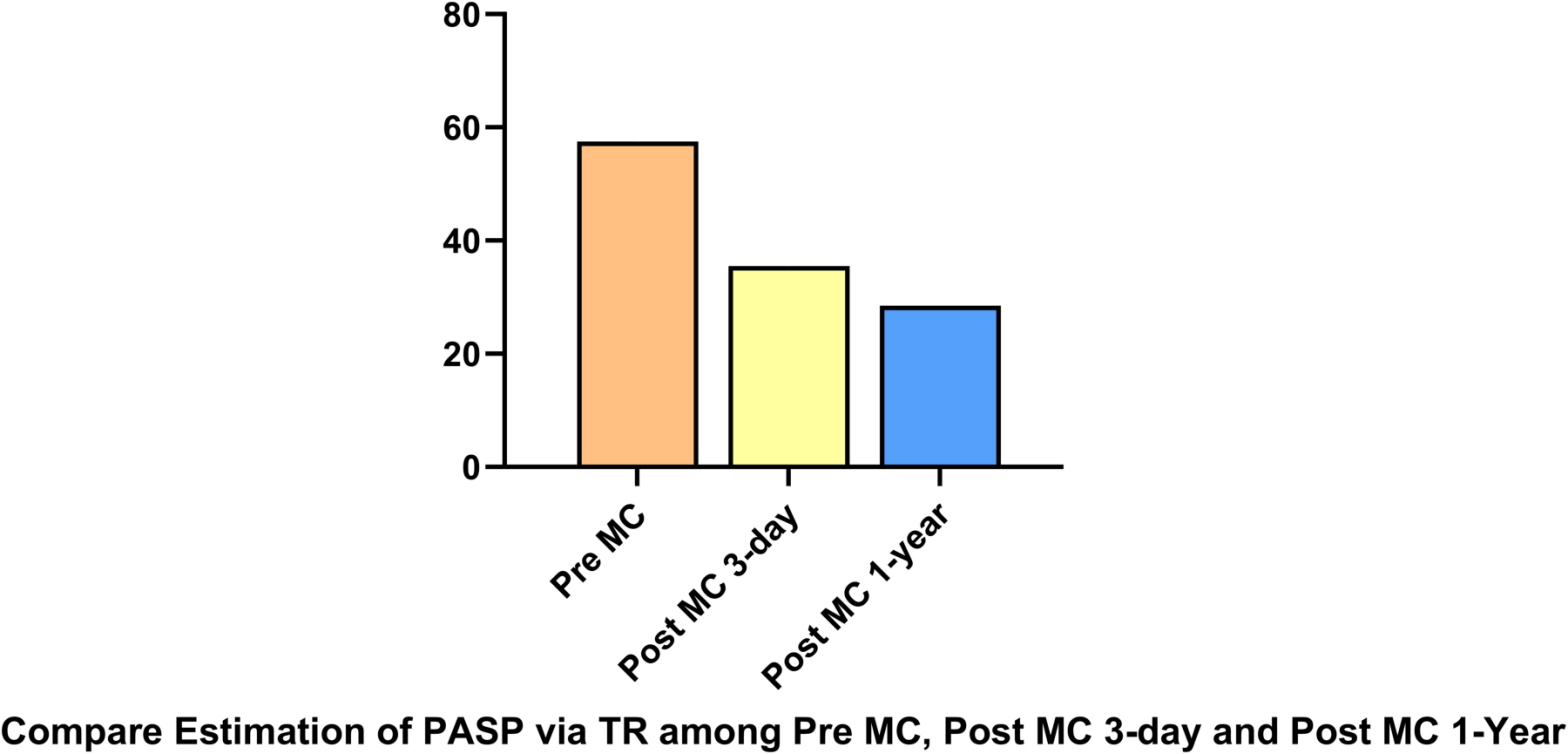
PASP via tricuspid regurgitation, demonstrating a significant reduction at both post-MC 3-day and post-MC 1-year compared to baseline.

LAP decreased significantly from 32.5 (14–48) mmHg pre-procedure to 19 (10–22) mmHg post-procedure. The MVA decreased from 3.9 (3.5–4.7) cm^2^ pre-procedure to 2 (1.8–2.5) cm^2^ post-procedure.MVA was measured pre-procedurally and immediately post-procedurally using 3D-TEE with MPR. At the 1-year follow-up assessment, the MVA was re-evaluated using three-dimensional transthoracic echocardiography (3D-TTE). PASP decreased from 56.5 (48–69) mmHg pre-procedure to 33.5 (33–42) mmHg post-procedure. NT-proBNP levels showed a downward trend (Figure 2). All patients demonstrated significant symptomatic improvement, with NYHA functional class improving from III–IV to I–II by the one-year follow-up, accompanied by the resolution of lower extremity edema. At the 3-day and 1-year follow-ups, all patients had MR graded as mild by transthoracic echocardiography. Four patients had only trivial residual MR. Quantitative assessment using the proximal isovelocity surface area (PISA) method confirmed a substantial reduction in regurgitant volume and effective regurgitant orifice area in each case compared to baseline. Mitral regurgitation was evaluated using comprehensive echocardiographic assessment (color Doppler, pulmonary vein flow, and quantitative measures) in accordance with current guidelines. Additionally, TTE was used to estimate PASP via tricuspid regurgitation, demonstrating a significant reduction at at both Post-MC 3-day.and Post-MC 1-year compared to baseline (Figure 3).

### Myocardial work and strain improvement

**Figure 4:**
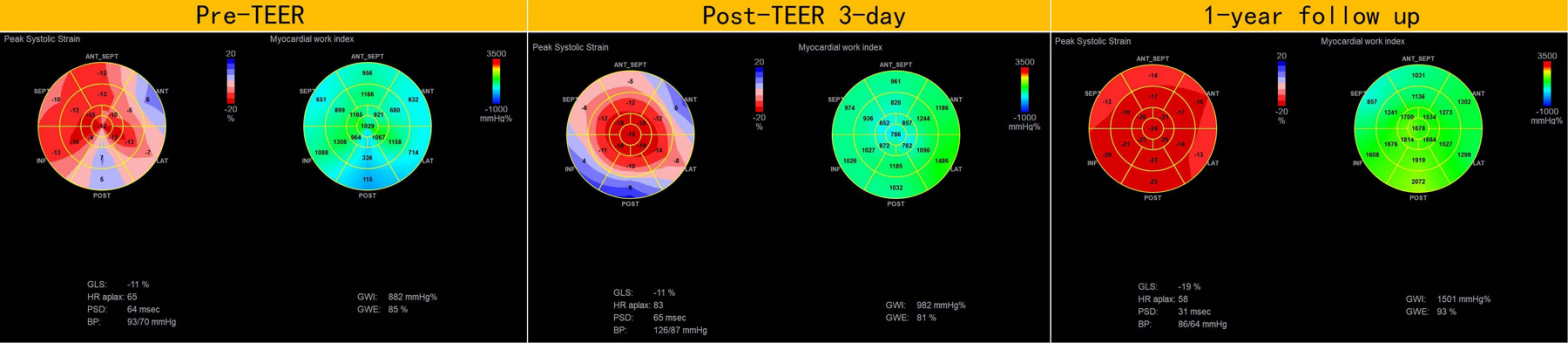
Compared to pre-MC, global longitudinal strain and myocardial work increased at both post-MC 3-day and post-MC 1-year, with statistically significant improvements noted at the 1-year follow-up.

**Figure 5:**
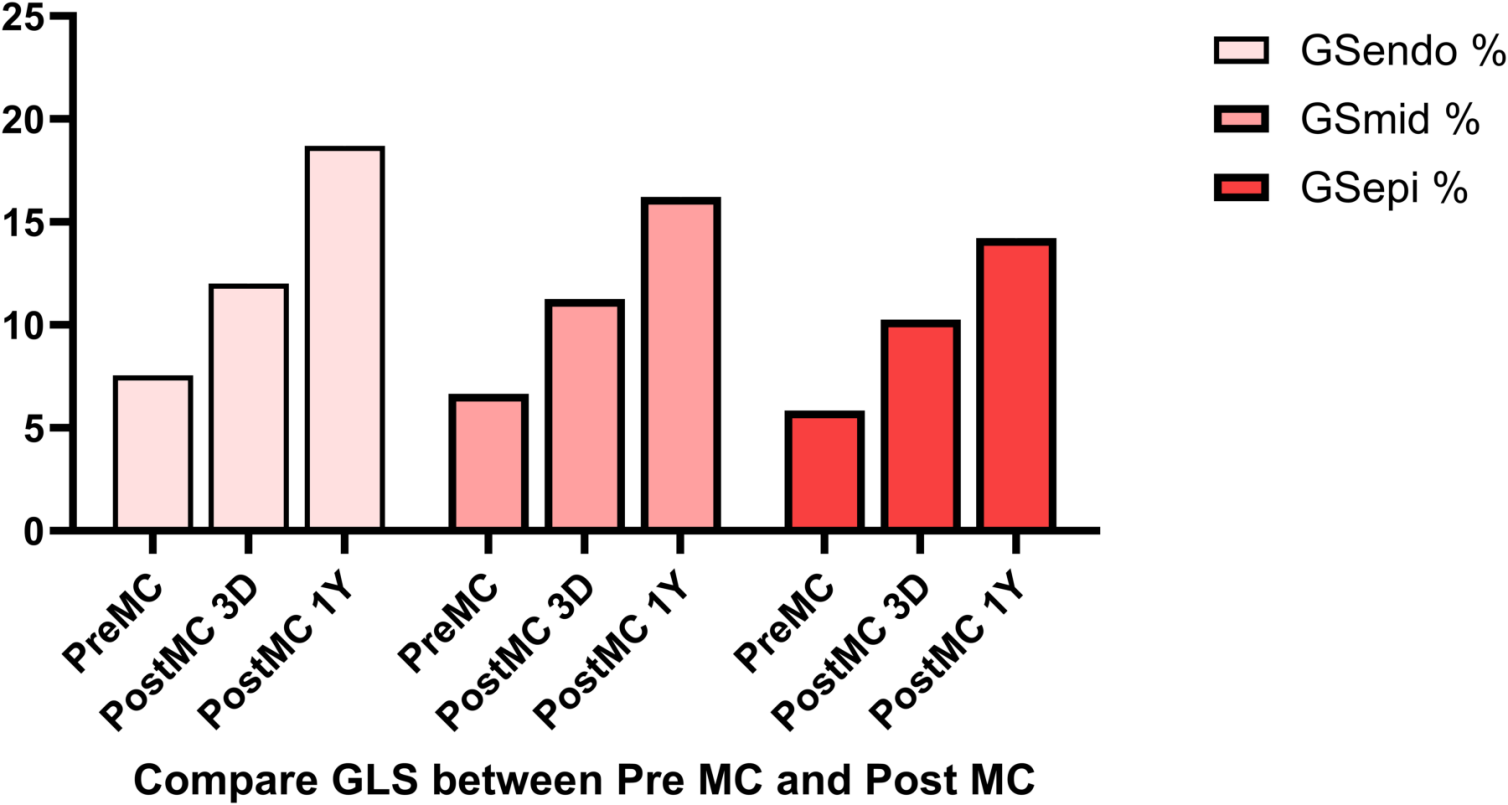
Global longitudinal strain (GLS) showed significant improvement post-procedure, with endocardial strain (GSendo) increasing from baseline to the 1-year follow-up. Mid-wall (GSmid) and epicardial strains (GSepi) also demonstrated significant improvements at the 1 year follow up.

**Figure 6:**
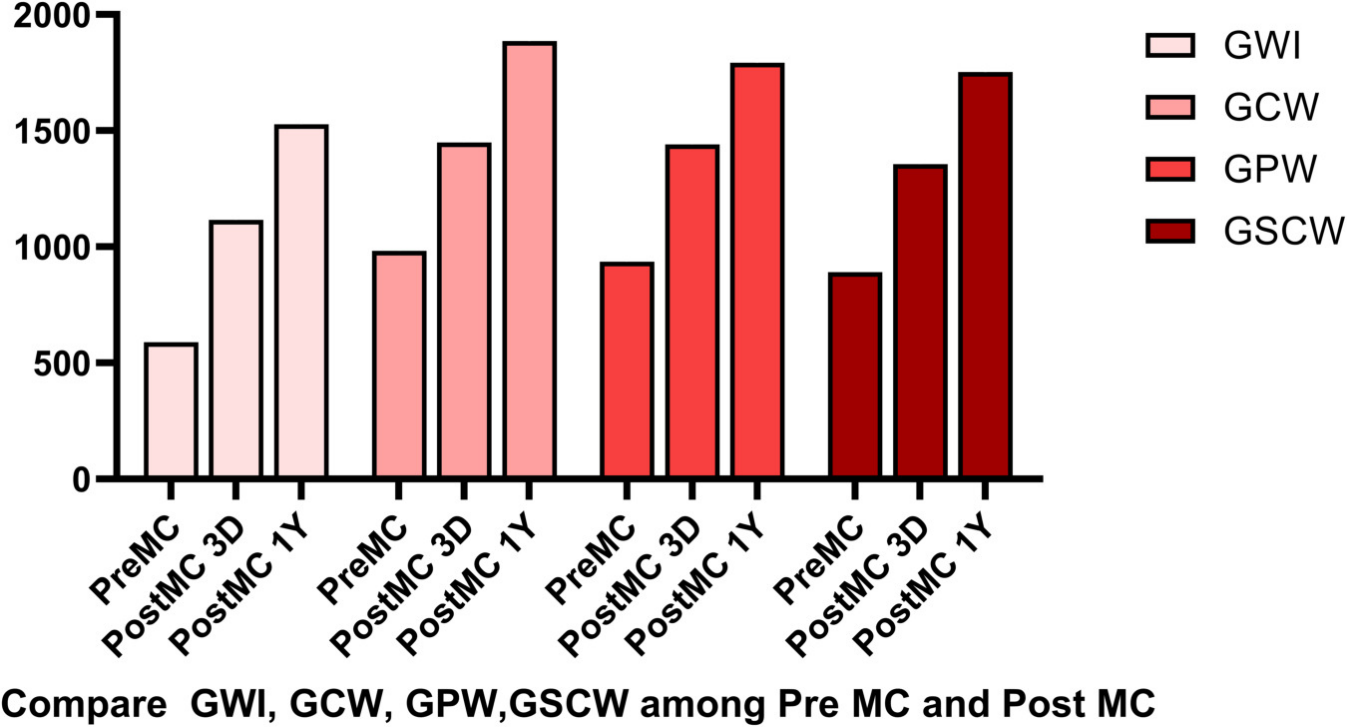
Compared to pre-MC, the global work index (GWI), global constructive work (GCW), global pressure-volume work (GPW) and global systolic constructive work (GSCW) increased at both post-MC 3-day and post-MC 1-year, with significant improvements noted at the 1-year follow-up.

**Figure 7:**
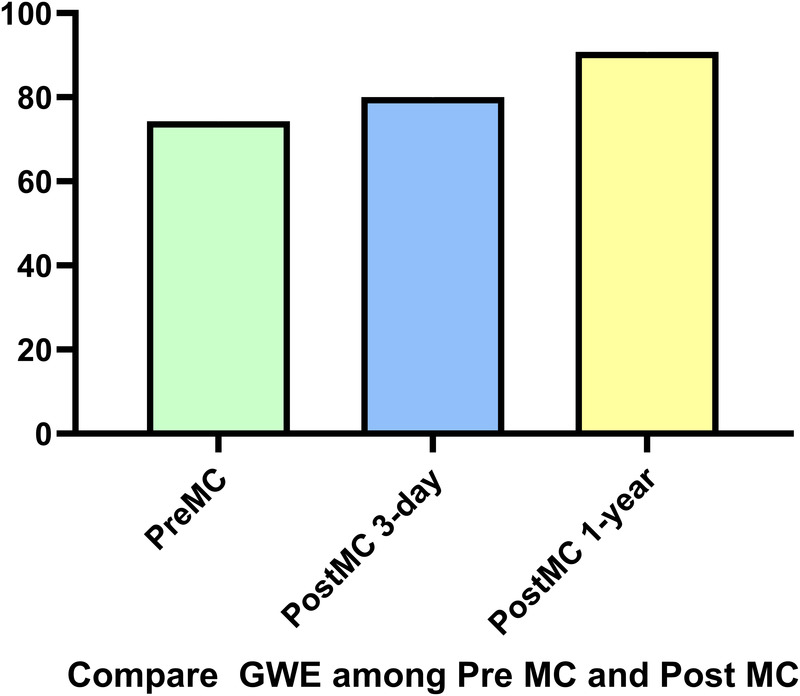
Compared to pre-MC, global work efficiency (GWE) increased at both post-MC 3-day and post-MC 1-year, with significant improvement noted at the 1-year follow-up.

**Figure 8:**
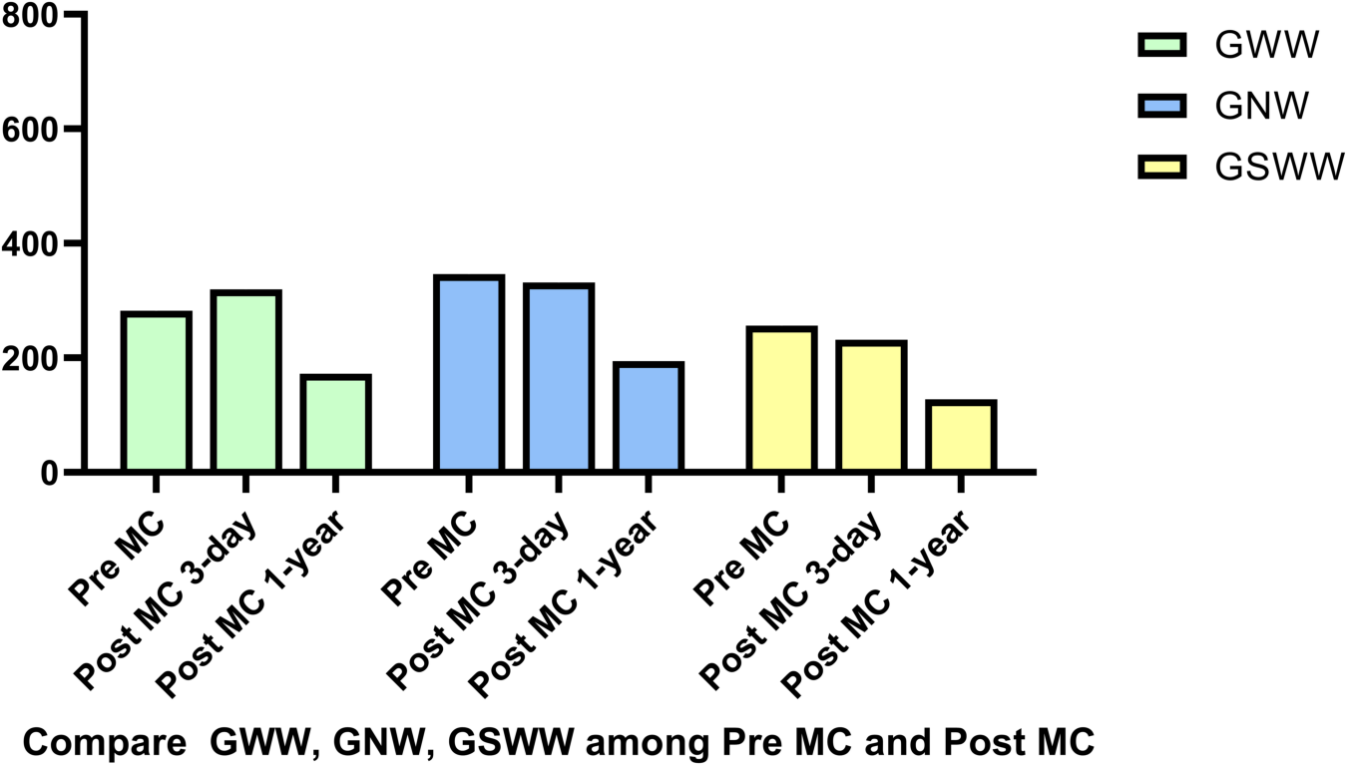
There were no significant changes in wasted work parameters, global wasted work (GWW), global systolic wasted work (GSWW) and global negative work (GNW).

In addition to the improvement in clinical symptoms, we observed evidence of reverse cardiac remodeling, indicated by favorable structural and functional changes in the left ventricle over the follow-up period. Although conventional echocardiographic parameters exhibited a trend toward reduction, these changes did not reach statistical significance (Table 3). Moreover, left ventricular global longitudinal strain (GLS) and myocardial work parameters showed significant improvements at the one-year follow-up. Endocardial strain (GSendo), mid-wall strain (GSmid), and epicardial strain (GSepi) all improved significantly compared to baseline. There were also improvements in the Global Work Index (GWI), Global Constructive Work (GCW), Global Pressure-Volume Work (GPW), and Global Work Efficiency (GWE) from three days post-procedure to the one-year follow-up. significant improvements were noted at the 1-year follow-up, indicating enhanced myocardial function and efficiency (Figures 4–7). No significant changes were observed in wasted work parameters, including Global Wasted Work (GWW) and Global Negative Work (GNW), over the follow-up period (Figure 8).

### Safety and complications

No major procedural complications were observed. TEER was well-tolerated in all patients, with no device-related complications such as clip detachment or significant bleeding. Follow-up confirmed the long-term durability of the repair, with no significant recurrent MR observed.

## Discussion

This case series highlights a unique and challenging anatomical variant of mitral valve disease characterized by an arcade-like mitral apparatus. This configuration, marked by thickened, shortened chordae tendineae and hypertrophied papillary muscles, makes surgical repair particularly challenging. Guidelines recommend prioritizing repair for primary mitral regurgitation (PMR) (6–8), as it preserves the native valve and avoids the complications associated with replacement. However, repair is often complex and time-consuming, leading many surgeons to opt for replacement instead (9). This approach, although simpler, results in significant challenges such as the requirement for lifelong anticoagulation in patients with mechanical valves and durability issues with bioprosthetic valves. These limitations highlight the shortcomings of current treatment practices and underscore the need for less invasive alternatives like TEER, particularly in elderly patients or those with significant comorbidities, where surgical risks are elevated. Our study demonstrated that TEER is a feasible, safe, and effective treatment option for managing severe mitral regurgitation in patients with arcade-like mitral apparatus.

The combination of thickened chordae, shortened leaflets, and an elliptical orifice requires careful procedural planning and imaging guidance to ensure adequate leaflet coaptation without inducing stenosis. In our series, a single XTR clip was used for all patients due to its suitability for the complex subvalvular anatomy, allowing sufficient leaflet grasp to reduce MR effectively while minimizing elevated transmitral gradients. The XTR's longer arm length provided versatility in capturing thickened, retracted leaflets, which is critical in patients with arcade-like mitral anatomy. Our decision was a practical one, dictated by real-world constraints of national device availability and our team's hands-on experience. This approach should be undertaken with caution and rigorous intraprocedural assessment of mitral valve area and gradient. Notably, at the time of this procedure, the fourth-generation MitraClip system (G4) was not yet available in China (it received local approval in April 2024), limiting our device choice to the third-generation clips. Although effective in select scenarios, a slow and controlled clip closure strategy represents a pragmatic compromise and may no longer be optimal given the availability of more advanced devices. The MitraClip G4 system offers enhanced leaflet control, such as independent gripper actuation and a broader range of clip sizes, features we would now favor to enable more precise and customized valve repair. Additionally, newer transcatheter edge-to-edge repair platforms such as the PASCAL P10 (Edwards Lifesciences) incorporate design elements like a central spacer and broad, contoured paddles, which help to distribute leaflet stress more evenly and may further reduce tension across the mitral valve. These advancements in device design underscore the rapidly evolving TEER landscape and inform a forward-looking procedural strategy, setting the stage for careful consideration of device selection in contemporary mitral repair.

Given the elliptical shape of the mitral orifice, maximizing leaflet coaptation without significantly reducing the valve orifice area was crucial. Coaptation was achieved with one patient in the lateral zone and the others slightly medial to the central zone, balancing MR reduction and valve orifice area maintenance. These findings expand the therapeutic scope of TEER in complex mitral anatomies, building upon previous studies (10, 11). Notably, all patients in our series presented with isolated MR and no evidence of mitral stenosis, indicating a normal-sized mitral annulus. This subset corresponds to the anomalous mitral arcade variant without stenosis (as originally described by Layman and Edwards, 1967) (1). Our results suggest that TEER is most feasible in this scenario. In cases of anomalous mitral arcade that present as a mixed lesion with both MR and MS (as reviewed by Hakim et al., 2013) (12), the smaller annular size and narrowed orifice could make TEER challenging or contraindicated due to the risk of creating a prohibitive transmitral gradient. In our patients, the effective valve orifice area decreased after clip placement [from 3.9 (3.5–4.7) cm^2^ to 2 (1.8–2.5) cm^2^], reflecting the Alfieri stitch effect of the MitraClip, yet the post-procedural transmitral gradients remained acceptable (all ≤5 mmHg). This implies that an adequate initial annular size allowed the clip to be deployed without inducing significant mitral stenosis.

Patients with an arcade-like mitral apparatus present unique anatomical challenges for TEER. This rare congenital anomaly of the subvalvular mitral apparatus is characterized by short, thickened chordae tendineae (often with direct insertion of the anterior leaflet into the papillary muscles) and a markedly reduced, elliptical valve orifice. Such distorted subvalvular geometry restricts leaflet mobility and alters coaptation, making it difficult to achieve a secure grasp of both leaflets. An elliptical (non-circular) mitral orifice and displaced papillary muscles further complicate device positioning, increasing the risk of suboptimal clip orientation or chordal entanglement. Indeed, prior experience has shown that TEER in patients with rheumatic or otherwise abnormal mitral valves (with thickened leaflets and chords) carries higher procedural complexity and complication rates (13), underscoring the need for meticulous planning in these cases.

To overcome these challenges, we employed advanced imaging guidance and tailored techniques. Real-time 3D TEE with live MPR was instrumental in delineating the abnormal anatomy and guiding precise clip positioning, as such complex repairs would not be feasible without 3D imaging guidance (14). We also adjusted the transseptal puncture site to optimize the trajectory for clip delivery; in our case the septum was punctured slightly posteriorly (and inferiorly) to achieve a more coaxial alignment with the mitral valve plane and adequate height above the valve (15).This strategic puncture location provided the catheter a favorable approach angle, helping the clip arms to open parallel to the valve coaptation line. During clip deployment, careful orientation under multiplane TEE visualization ensured the clip crossed the elliptical orifice at the optimal location to capture both leaflets while avoiding the thick chordae.

Through these measures, we achieved an effective reduction of mitral regurgitation despite the complex arcade-like anatomy. Our experience demonstrates that, with meticulous imaging guidance and technique, TEER can be successfully applied even in anatomically challenging mitral valves that have traditionally been considered unsuitable for catheter-based repair.

However, it should be noted that this approach is applicable to adult patients with isolated MR. In infants or young patients—who often present with anomalous mitral arcade accompanied by mitral stenosis-surgical repair or replacement remains the definitive treatment, as catheter-based interventions are not feasible in those scenarios.

We acknowledge that our case series is limited by the very small sample size (*n* = 4) and the relatively short follow-up period (one year). These limitations mean that the findings should be interpreted with caution. Future research should aim to include a larger patient cohort and extend the follow-up period to confirm the long-term durability of TEER in this unique population.

## Conclusion

In conclusion, TEER is a feasible and effective therapeutic option for high-risk patients with severe MR secondary to an arcade-like mitral apparatus. This study provides valuable data regarding the use of TEER in a rare and anatomically complex population, supporting its role as an alternative to traditional surgery in anatomically challenging cases. Future research should aim to expand the patient cohort and extend the follow-up period to confirm the long-term durability of TEER in this unique population.

## Data Availability

The raw data supporting the conclusions of this article will be made available by the authors, without undue reservation.
