# Supplementary figures and images for: Transcatheter edge-to-edge repair for arcade-like mitral apparatus: expanding treatment options and assessing clinical outcome
Source: Front Cardiovasc Med. 2025 Sep 1;12:1571818. doi: 10.3389/fcvm.2025.1571818 (PMC12433959; doi:10.3389/fcvm.2025.1571818)

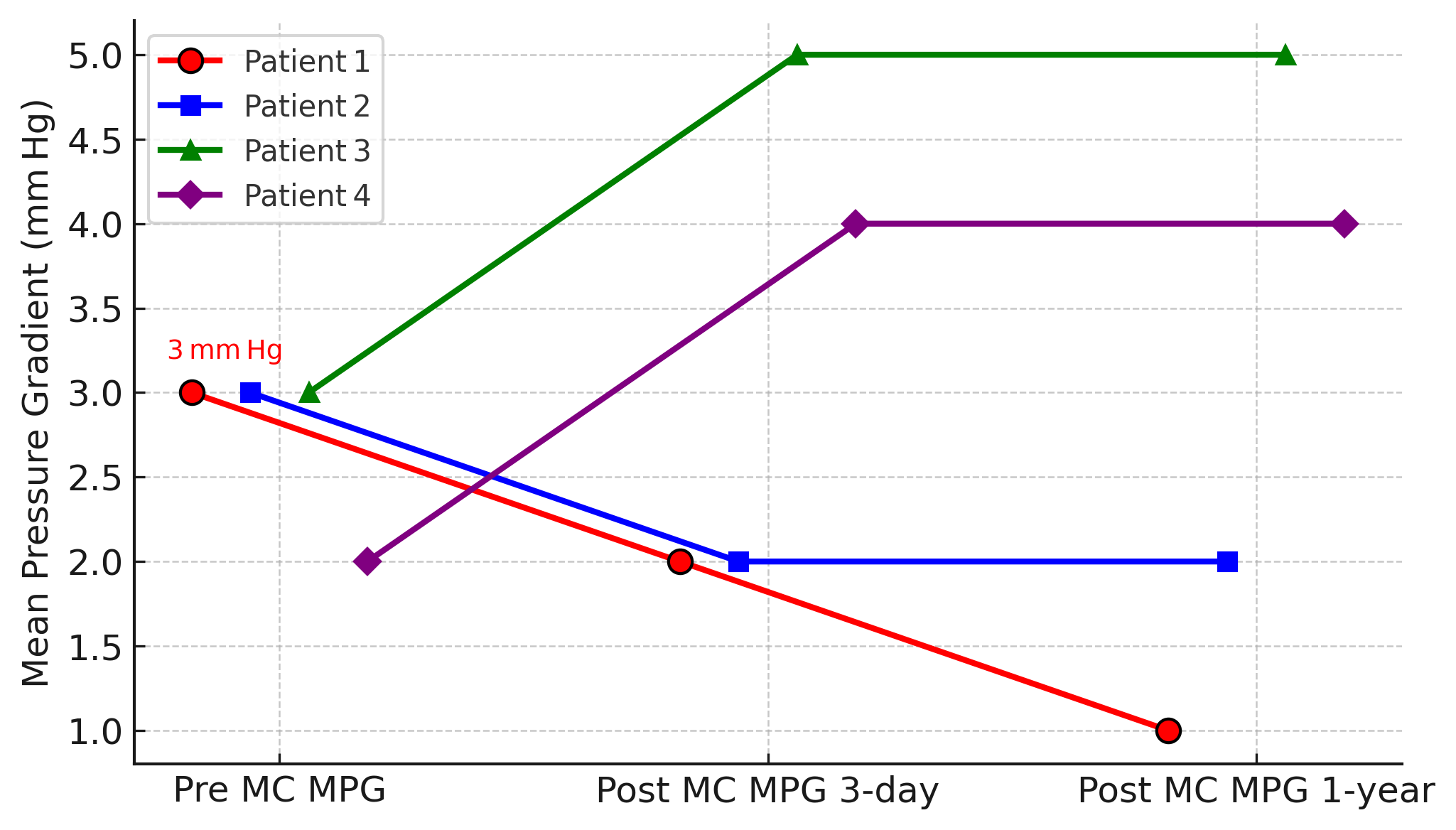

Supplement: Supplementary file 1 [file Image1.png]
